# Some Account of Erysipelatous Fever as It Occurred in Whitesburg and Its Vicinity, Madison County, Ala., during the Spring and Summer of 1844

**Published:** 1845-07

**Authors:** Preston Capshaw

**Affiliations:** Whitesburg, Ala.


### THE WESTERN JOURNAL OF MEDICINE AND SURGERY.

JULY, 1845.

Art. I.—*Some account of Erysipelatous Fever as it occurred in Whitesburg and its vicinity, Madison county, Alabama, during the Spring and Summer of* 1844. By Preston Capshaw, M.D., of Whitesburg, Ala.

Whitesburg is situated eleven miles south of Huntsville, on the north bank of the Tennessee river. It is a place of little importance, except as a landing for *flat-boats*, and a point for the shipment of cotton, &c., to New Orleans.

The country in the vicinity, is diversified—on the east we have a ridge of mountains making in towards the river, while to the north and west are a few scattering spurs and knobs rising from a general plain but little elevated above the banks of the river. This plain is so much cut up with ponds and sloughs, as to have given currency to the local appellation of *Pond Beat.* During high tides in the river about one sixth of the surface is subject to inundation. The lands not occupied by mountains and free from overflow are generally of good quality, and mostly reduced to cultivation. The inhabitants number about 900, two-thirds of whom are slaves, employed in the production of cotton. The prevailing diseases are of malarious origin, and *chills* are so common, that few are so fortunate as to escape them a whole year.

*Geological position.*—The diluvial surface is underlayed by the common grey cherty limestone of the carboniferous system. It also occupies the base and body of the mountains, and seems to rest in a horizontal position. The mountains are capped with *outliers* of sandstone (intermixed in places with conglomerate water-worn pebbles) which varies in thickness from 50 to more than 200 feet, and probably *dips* a few degrees eastward.

The limestone abounds in fossil remains, among which are species of the Cyathophyllum, Columnaria syringipora, Encrinites, Pentacrinites, Platycrinites, Pentremites, in abundance, Terebratula, Productata, and a great many others common to this system. In the sandstone we find Lepidodendra, Calamites, Sigillaria, &c., with seams of bituminous coal, and fossil charcoal.

The botanical productions differ in nothing material from other places similarly situated, in the same latitude.

With this brief preface, I shall proceed at once to the object of my communication.

About the 20th of March, a negro woman, belonging to Mr. Edwards at Whitesburg, was attacked with erysipelatous inflammation of the face and head, attended with high fever, sore throat, and other symptoms characteristic of this form of disease. She had been in the service of Mr. Edwards only a few days when attacked, and did not know when or how she contracted the disease. Mr. Edwards attended to the administration of medicines to the negro woman, and was himself attacked about the first day of April. His affection was much like the negro's. On the 13th or 14th of April, three children at Mr. Hill's were simultaneously attacked. On the Sunday preceding their attack, (just a week) they had visited Mr. Edwards', and were in the rooms occupied by both Mr. E. and his negro. On the 20th, Warren, a mulatto boy, who stayed a good deal in the house at Mr. Hill's, was attacked. On the 28th, Harriet, a negro, who attended in the house, was attacked; also William, a brother to Warren, was attacked on the same day. On the 29th. Mr. Corlew, then residing with Mr. Hill, was attacked. The last four cases occurred in persons who had been constantly with the sick children till attacked themselves. About the 5th or 6th of May, Miss McLeod, a sister to Mrs. Hill, was attacked—previous to her attack she had spent eight or ten days in watching and assisting to nurse her sister's children. At the commencement of her attack she returned to her brother's, with whom she resided, sent his children from home, and debarred all visitors, except her physician and nurse. In about two weeks her fever abated, and she was so much better, that the children were suffered to return home. On the 23d, some four or five days after her return home, a little daughter of Mr. McLeod, was attacked, and on the 28th an old negro woman who waited on Miss McLeod throughout her illness, also suffered an attack of the disease; and on the 1st of June two negro children, belonging to the same, and who had been sent off with the white children, were attacked. On the 1st of June, the disease appeared in the family of a quatroon washerwoman. She had done the washing for Mr. Hill's family since the 5th of May. The first case occurred in a little boy about three years old. About the first of August, the woman and another son were attacked; and about the first of September, two daughters of the same were attacked; thus accomplishing the family circle. From these facts it would seem that not a single case occurred except in persons who visited or had communication with the sick, though it must be acknowledged that a greater number escaped than suffered from such intercourse.

The total number of cases was 19—of which 7 occurred in whites, 5 in negroes, and 7 in persons of mixed blood; mulattoes, quatroons, &c.: 12 cases occurred in children mostly under 10 years old, and the remaining 7 were persons over 30, and mostly of delicate and susceptible constitutions With regard to sex, 8 were males, and 11 females.

Out of the total number of cases, but one terminated in death. It occurred in a negro woman of delicate constitution, laboring under erysipelas of the face and head. She died of *asthma* on the 9th day from her attack; the pulse ceasing to be felt at the wrist 24 hours before death. The period of recovery occupied from two weeks to a month, and in some cases a still longer time.

For convenience of illustration, I shall arrange the cases in three *groups*, corresponding to the seat of the local affection.

1st. Cases in which the inflammation occupied the face and head.

2d. Cases in which the inflammation occupied the lower extremities.

3d. Cases in which the inflammation occupied the throat, without appearing on the skin.

Belonging to the first group were 11 cases; to the second 3; and to the third 5.

I will now introduce a single case from each of these groups, with such additional remarks as may seem necessary to convey a tolerably accurate idea of the disease.

Group 1st.—*In which the inflammation occupied the face and head:*

Case. Elizabeth, daughter of Mr. Hill, about 6 years old, was attacked on the 13th of April with something like a chill, followed by fever, pain in the head and neck, hoarseness, sore throat, &c. On the 14th her condition was much the same, except that she complained more of her throat, and experienced some embarrassment in breathing. Towards evening her breathing became stridulous, which increased to such a degree in the course of the night that suffocation seemed inevitable. On the morning of the 15th these distressing symptoms had in a great degree subsided, and she appeared more at ease, though the fever continued with but little abatement. Towards evening she complained much of pain in the nose and across the face, but no swelling or redness was apparent. On the 16th all distress attending respiration had pretty well passed off, but her fever continued as before, and her nose and face were swollen, as from the sting of a bee. On the 17th the inflammation had spread over the whole face as far back as the ears, closing the eyes, and disfiguring and disguising the whole countenance. The fever still continued without intermission—the pulse ranging between 120 and 130. On the 18th, the face swollen as before, and scattered over with vesicles filled with a darkish serous fluid. On the 19th, inflammation subsiding at the place of commencement, but still spreading over the scalp and neck; fever without intermission. On the 20th, the inflammation still declining; detached cuticle falling off; inflammation has spread but little since yesterday; fever seems also on the decline. On the 21st, the inflammation and fever still declining, and the patient decidedly better. From this time she continued regularly to improve, and was well in about three weeks from the date of her attack.

In this case occurred the most decided stridulous symptoms of anyin the present group, though all were alike affected, at the commencement, with sore throat, oppression about the chest, and difficult or distressed breathing. The upper part of the throat had a red œdematous appearance, but attended in no instance with ulceration or sloughing. I consider the foregoing case about a fair sample of its class, though several of them were of a more grave character, and of more protracted duration. Convalescence in some cases was attended with troublesome sores about the scalp, and in one case a blister to the chest put on a very angry appearance. Some cases were attended with coma; in the above there was but little, and in others none at all. Some of the cases were likewise attended with delirium.

Group 2d—*In which the inflammation occupied the lower extremities:*

Case. Warren, a mulatto boy, about seven years old, attacked about the 20th of April. He first complained of severe pain in the top of the right foot, together with some febrile excitement. On the 21st, his foot was swollen and very painful, attended with increase of fever; but no cough or sore throat. On the 22d, the foot and ancle were much swollen, very painful, and covered with numerous small blisters. On the 23d, the inflammation extended about 4 inches above the ancle joint, after which it progressed more rapidly, attended with high fever, and frequent, rapid pulse, till it reached the inferior angle of the scapula in an upward direction. It then passed over to the left side, and pursued a retrogade direction down the left leg, as far as the ancle, when it subsided without involving the foot. The period occupied in traversing the above described surface was about two weeks. There also formed, about the point of commencement, a troublesome abscess, which discharged a great deal of *matter*, and the boy was left in such a debilitated condition, that he was probably two months in regaining the powers of *self-transportation.*

I have to remark concerning this group, that two out of the three cases, were more protracted than any others. The other was a mild case, and the only one of the group that was attended with sore throat, or embarrassment of the respiratory organs. A hot, dry skin, and frequent pulse, was generally maintained throughout the course of the disease, which subsided with the inflammation, leaving the patients much debilitated.

Group 3d—*In which the throat was affected without inflammation of the skin:*

In illustration of this group I will introduce the case of my late friend, Dr. Russell, of Huntsville; for the report of which I am indebted to Dr. Erskine, his late partner, and principal attending physician during his illness.

Dr. Russell possessed a firm constitution, and had never been seriously ill before in his life. Relying on the powers of his constitution for the eradication of this disease, which did not seem at first of a serious character, he neglected to take medicines, though urged by his physicians to do so, until it was too late. I give the case in the words of Dr. Erskine.

"On the 12th and 13th of May, 1844, Dr. Russell visited Miss McLeod, laboring under an erysipelatous affection of the neck, face, and throat. About the middle of June Miss McLeod visited Dr. R.'s in Huntsville, and spent several days at his house. She was still much debilitated by her former attack, and suffered from derangement of the bowels and some fever, which passed off in a few days without much treatment. Shortly after this, a servant girl who waited on Miss McL. was taken with sore throat accompanied with neuralgic pains about the face and neck, which yielded in 8 or 10 days to mild treatment. One or two other servants were siezed with similar symptoms, all of which passed off under the use of emetics, cathartics, sinapisms, quinine and gargles.

"About the 8th of July Dr. Russell complained of slight sore throat with neuralgic pains about the neck and face, and some febrile disturbance.

"Although impressed with the belief that he labored under the same disease which had affected his servants, yet he used no treatment except some mild gargles, and continued at business until Wednesday, the 10th, when he had a regular paroxysm of fever, ushered in by a distinct chill. The fever passed off in 8 or 10 hours with free perspiration. During the paroxysm the throat became more painful with increase of hoarseness, all of which passed off with the fever. On the 11th and 12th, until 4 o'clock in the evening, he remained free from fever, and took quinine though not freely. Another chill then came on followed by high fever, with considerable increase of the local affection. The erysipelatous affection appeared to have traveled all over the pharynx and the upper part of the larynx, exciting an unpleasant cough. The fever passed off as before, yet the local affection continued very troublesome, and the cough distressing, emitting something of a croupy sound. On Sunday morning, the 13th, he was very restless, and discharged quantities of thick ropy phlegm; there was also hoarseness and more of the croupy sound in coughing. About 4 or 5 o'clock on Sunday evening there was an evident increase of fever without any perceptible chill, and with it a great increase of hoarseness, and cough, with general oppression about the chest, and a great sense of suffocation. These symptoms continued to increase during the night, being occasionally much worse, as if some spasmodic action had been induced. He urged his physicians in the event of suffocation to perform the operation of laryngotomy. These spasmodic paroxysms about the lungs increased both in frequency and violence until between 9 and 10 o'clock on Monday morning, when he literally suffocated. The operation desired was performed, but without affording him any relief. Dr. R. frequently remarked some 15 or 20 hours before his death, and up to that period, that he could not fill his lungs during inspiration; and from the fact that no relief was afforded by the operation, we have no doubt, but that the inflammation had extended throughout the bronchia. Dr. R. died with all the symptoms of confirmed croup."

Among Dr. R.'s servants I understood 3 cases occurred, in one of which erysipelatous inflammation traversed the face, neck, and scalp—the throat was affected in the other two.

In remarking on Dr. R.'s case I must acknowledge, that it differs somewhat from those that occurred under my own observation. It is true, that nearly every case within my knowledge was attended, at the commencement, with some feeling of rigors, and some of them with tolerably distinct chill, but I do not recollect a single instance in either form of the disease, in which it assumed anything approaching to the intermittent form. I observed the pulse closely throughout the inflammatory stage, in cases belonging to each group, and unless I am greatly mistaken, it did not vary more than ten beats at any period within the 24 hours.

Again—I observed nothing of the intermitting spasmodic action about the lungs referred to in the above case, though all in the present, and a large number in the first group, had much oppression about the chest, with anxious respiration, and many of them gave the croupy sound in coughing. In other respects the case differs but little from those of the same character observed by myself—except in its termination.

I consider this last decidedly the mildest, though perhaps not the least dangerous form of the disease. It generally ran its course in from six to ten days, and the patient recovered without that state of extreme debility which attended recovery from the other forms.

The fever attending this disease in all its forms, was characterized by a frequent, and rather contracted pulse, a dry, hot skin, generally great thirst; tongue coated thickly with a whitish or brownish fur, and in all respects the fever approached more nearly our *typhoid or winter fevers*, than any other form with which I am acquainted. It always preceded the inflammation of the skin a day or two, while in the throat cases, the inflammation and fever seemed to be developed together. The fever and inflammation generally subsided together, though in some cases the febrile excitement in some degree, lingered a few days longer.

*Treatment.*—In the early stages, and in cases attended with sore throat, and oppressed and impeded respiration, *emetics*, repeated according to the urgency of the symptoms, were of decided benefit. In some of the most urgent cases, they were repeated as often as three or four times in 24 hours, and always with evident relief to the patient. The only articles of this class used were ipecac. and compound syrup of squills.

*Bloodletting* was practiced in but a single instance. The patient did well, but owing to the frequency and smallness of the pulse, I regarded it as a remedy of doubtful utility, and did not venture to try it again.

*Purgatives.*—In some of the first cases, I employed calomel, either alone or in combination, but a slight ptyalism having occurred, causing an aggravation of the other symptoms,

I abandoned it, and substituted sulph. magnesia or jalap, either of which answered a valuable purpose—that of promoting, when desired, a free evacuation of the bowels.

*Tonics.*—The only article used with this view was quinine. It was administered in all stages of the disease, but never with decided benefit, unless perhaps in the stage of convalescence.

*Blisters.*—These were decidedly the most valuable auxiliaries in the treatment of cases attended with oppressed and difficult breathing, and sore throat accompanied with croupy symptoms. I think I saw several cases which must have died, had it not been for blisters and a liberal use of emetics.

*Other topical remedies.*—Of those used for the purpose of arresting the progress of the inflamed surface, I can only say, that I have no confidence in any. I tried stimulating and astringent lotions; drew caustic lines around and about the inflammation, and raised blisters in front of it, all, however, without checking "the even tenor of its way." Cold lotions of water, or water and vinegar, or salt water, applied by means of cloths laid over the part, seemed soothing and comfortable to the patient, and probably exercised some influence over the inflammation, as far as the application of cold was concerned.

*Gargles* of every description, I look upon as perfectly useless in this disease, except to cleanse the mouth, and afford a species of amusement to the patient. The mischief lies beyond their reach.

With this brief view of the principal facts and circumstances connected with the disease, as it presented itself to my observation, I leave the reader to his own comments, and free to arrive at such conclusions as will most naturally arise from its careful perusal.

May 3d, 1845.

